# Crystal structure of aceto­nitrile­[η^6^-1-methyl-4-(1-methyl­eth­yl)benzene][1-(pyrimidin-2-yl)-3*H*-indol-1-ium-2-yl-κ^2^ *N*,*C*]ruthenium(II) bis­(hexa­fluorido­anti­monate)

**DOI:** 10.1107/S2056989015016710

**Published:** 2015-09-17

**Authors:** Carina Sollert, Andreas Orthaber, Lukasz T. Pilarski

**Affiliations:** a Uppsala University, Department of Chemistry – BMC, Box 576, 75123 Uppsala, Sweden; b Uppsala University, Department of Chemistry – Ångström Laboratories, Box 523, 75120 Uppsala, Sweden

**Keywords:** crystal structure, cyclo­metalated Ru^II^, pyrimidyl-3*H*-indole, *para*-cymene, C—H⋯F hydrogen bonds

## Abstract

In the title compound, [Ru(C_10_H_14_)(C_12_H_9_N_3_)(CH_3_CN)][SbF_6_]_2_, the ruthenium(II) cation is *η* ^6^-coordinated by the *para*-cymene ligand with a Ru–centroid(*η* ^6^-benzene) distance of 1.746 (2) Å. Furthermore, ruthenium coordinations to the C and N atoms of the pyrimidyl indole ligand are found to be 1.986 (4) and 2.082 (3) Å, respectively. The typical piano-stool coordination environment is saturated with an aceto­nitrile solvent mol­ecule with a Ru—N distance of 2.044 (3) Å. The indolyl ligand is protonated at the C3 position with the N=C imine bond length appropriate to that of related 3*H*-indole-based complexes. In the crystal, the complex cation is linked to the SbF_6_^−^ ions through weak C—H⋯F hydrogen bonds.

## Abstract 2

Cyclo­metalated Ru complexes play a major role in catalytic transformation. The Ru cation is coordinated by a pyrimidyl-3*H*-indole ligand, as well as a *para*-cymene ligand and one aceto­nitrile mol­ecule.

## Chemical context   

**Figure 1:**
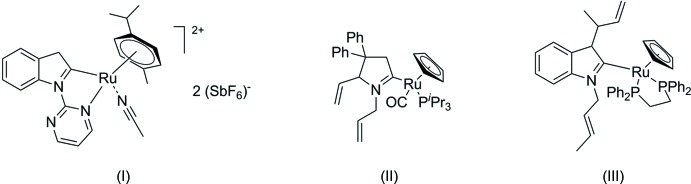
The title compound (I) and related cyclo­metalated ruthenium complexes (II) (Sollert *et al.*, 2015 ▸) and (III) (Chiang *et al.*, 2010 ▸).

Cyclo­metalated ruthenium compounds are well known catalytic inter­mediates in the C—H activation of various substrates (Arockiam *et al.*, 2012 ▸; Li *et al.*, 2012 ▸; Ferrer Flegeau *et al.*, 2011 ▸). In a recent study on oxidative Ru-catal­ysed heteroarene C—H aryl­ation (Wang *et al.*, 2015 ▸; Ackermann & Lygin, 2011 ▸), we demonstrated that [{RuCl_2_(*p*-cymene)}_2_] in the presence of AgSbF_6_ selectively ruthenates the C2—H bond of *N*-pyrimidine-substituted pyrroles and indoles (Sollert *et al.*, 2015 ▸). We concluded that in our catalytic system, the resulting ruthenacyclic species likely act as precursors rather than on-cycle inter­mediates. In the course of our studies we observed the unusual formation of the title complex, which shows protonation at the C3 position. The title compound and related cyclometalated ruthenium complexes are shown schematically in Fig. 1 ▸.
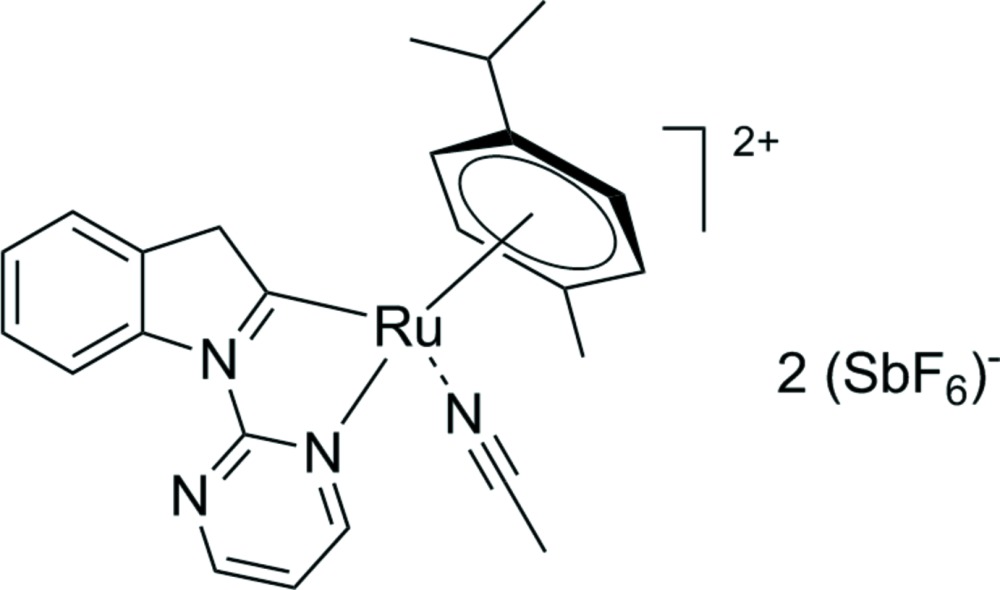


## Structural commentary   

**Figure 2:**
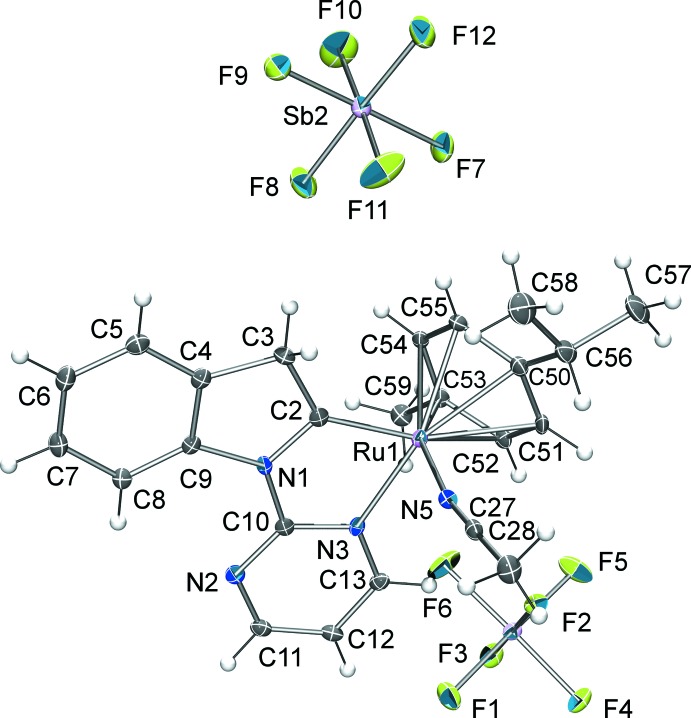
*ORTEP* representation of the mol­ecular components of the title compound, showing 50% probability displacement ellipsoids.

In the title compound (Fig. 2 ▸), the ruthenium(II) cation is coord­inated in an η^6^ fashion by a *para*-cymene unit. The Ru—C_*p*-cymene_ distances range from 2.197 (4) to 2.298 (4) Å. The centroid of the *para*-cymene benzene ring (*Cg*) shows an Ru1—*Cg* distance of 1.746 (2) Å. Furthermore, ruthenium coordinations to C2 and N3 of the pyrimidyl indole are found to be 1.986 (4) and 2.082 (3) Å, respectively. The coordination environment is saturated with one aceto­nitrile solvent mol­ecule, with an Ru1—N5 distance of 2.044 (3) Å. The least-squares planes of the 3*H*-indole ring system [r.m.s. deviation = 0.026 (4) Å] and the pyrimidine heterocycle [r.m.s. deviation = 0.013 (4) Å] are almost co-planar, making a dihedral angle of 2.6 (2)°. The Ru atom deviates by only 0.056 (1) Å from the 3*H*-indole plane. The 3*H*-indole shows a clear C2—N1 double bond of 1.345 (5) Å in the typical range for this class of compounds. The coordinating aceto­nitrile solvent mol­ecule shows slight deviation from a linear arrangement [C27—N5—Ru1 = 170.4 (3)°].

## Supra­molecular features   

The packing allows no direct inter­action of equivalent ruthenium complexes. The crystal packing shows a complex pattern in which two crystallographically independent SbF_6_^−^ counter-ions occupy a void formed by symmetry-equivalent metal complexes. C—⋯H hydrogen bonds of the pyrimidyl­indole and *para*-cymene ligands with the SbF_6_^−^ ions mainly account for the observed packing pattern (Table 1 ▸).

## Database survey   

This structure is related to chloro­(η^6^-*para*-cymene)[κ^2^-*N,C*-1-(pyrimidin-2-yl)-1*H*-indole]­ruthenium (Sollert *et al.*, 2015 ▸), in which the double bond is at C2=C3. The Ru1—C2 and Ru1-cymene distances, however, are almost unaltered. This is consistent with the development of a positive charge at N1 to effect the C3 protonation rather than at the Ru^II^ atom. The C2 atom in the title compound is therefore formally an anionic ligand, and not a carbene carbon. A similar cyclo­metalated pyrrolinyl complex (2) Buil *et al.*, 2015 ▸; Fig. 1 ▸) was obtained through HBF_4_-mediated rearrangement of *N*-allylic substituents. The Ru—C distances of 2.077 (4) Å (Buil *et al.*, 2003 ▸) are comparable to the Ru1—C2 distance of the title compound. The Ru-catal­ysed rearrangement of a 1,7-eneyne afforded the C2-cyclo­metalated 3*H*-indole (3) (Chiang *et al.*, 2010 ▸; Fig. 1 ▸). Structural parameters of this cyclo­penta­dienyl-coordinated ruthenium complex are in good agreement with the title compound.

## Synthesis and crystallization   

A pre-dried Young’s tube was charged with chlorido­(η^6^-*para*-cymene)[κ^2^-*N,C*-1-(pyrimidin-2-yl)-1*H*-indole]­ruthenium (50 mg, 1.0 equiv., 0.11 mmol) and AgSbF_6_ (76 mg, 2.0 equiv., 0.22 mmol). The tube was evacuated and backfilled with argon three times. The tube was equipped with a rubber septum and anhydrous MeCN (2 mL) was added *via* a syringe. The septum was removed, the tube sealed and wrapped in aluminium foil to protect the reaction mixture from light. The mixture was left stirring at room temperature for 18 h, after which the resulting precipitate was filtered off rapidly under air and the filtrate transferred immediately into a pre-dried round-bottom flask under argon. The solvent was evaporated under reduced pressure and a green solid was obtained. The solid was dissolved in d_8_-THF and transferred into a NMR tube under argon. The title compound was obtained as green crystals upon slow evaporation of the solvent.

## Refinement   

Crystal data, data collection and refinement details are summarized in Table 2 ▸. All H atoms on carbon were placed at calculated positions [C—H = 0.95 (aromatic), 0.98 (meth­yl), 0.99 (methyl­ene) and 1.00 (methine) Å] using a riding model with *U*_iso_(H) = 1.2*U*_eq_(C) or 1.5*U*_eq_(C_meth­yl_). The Ru—C bonds were ignored in the ideal placement of the aromatic H atoms.

## Supplementary Material

Crystal structure: contains datablock(s) I, global. DOI: 10.1107/S2056989015016710/is5413sup1.cif

Structure factors: contains datablock(s) I. DOI: 10.1107/S2056989015016710/is5413Isup2.hkl

Supporting information file. DOI: 10.1107/S2056989015016710/is5413Isup3.mol

CCDC reference: 1027878

Additional supporting information:  crystallographic information; 3D view; checkCIF report

## Figures and Tables

**Table 1:** Hydrogen-bond geometry (, )

*D*H*A*	*D*H	H*A*	*D* *A*	*D*H*A*
C11H11F7^i^	0.95	2.54	3.398(6)	151
C12H12F1	0.95	2.39	3.157(5)	138
C13H13F2	0.95	2.30	3.229(5)	167
C51H51F11^ii^	0.95	2.54	3.485(6)	174
C52H52F2	0.95	2.50	3.337(5)	147
C54H54F5^iii^	0.95	2.26	3.110(5)	148
C59H59*B*F6	0.98	2.32	3.253(6)	158
C59H59*C*F5^iii^	0.98	2.45	3.270(6)	141

Symmetry codes: (i) 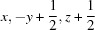; (ii) 
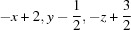
; (iii) 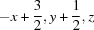.

**Table 2:** Experimental details

Crystal data	
Chemical formula	[Ru(C_10_H_14_)(C_12_H_9_N_3_)(C_2_H_3_N)][SbF_6_]_2_
*M*_r_	943.06
Crystal system, space group	Orthorhombic, *P* *b* *c* *a*
Temperature (K)	100
*a*, *b*, *c* ()	16.6046(8), 15.5955(7), 23.2786(12)
*V* (^3^)	6028.2(5)
*Z*	8
Radiation type	Mo *K*
(mm^1^)	2.37
Crystal size (mm)	0.18 0.17 0.08

Data collection	
Diffractometer	BrukerAPEXII with CCD
Absorption correction	Multi-scan (*SADABS*; Sheldrick, 1996 ▸)
*T*_min_, *T*_max_	0.578, 0.746
No. of measured, independent and observed [*I* > 2(*I*)] reflections	27645, 6643, 4920
*R*_int_	0.054
(sin /)_max_ (^1^)	0.643

Refinement	
*R*[*F* ^2^ > 2(*F* ^2^)], *wR*(*F* ^2^), *S*	0.033, 0.074, 1.01
No. of reflections	6643
No. of parameters	392
H-atom treatment	H-atom parameters constrained
_max_, _min_ (e ^3^)	0.89, 1.00

Computer programs: *APEX2* and *SAINT* (Bruker, 2012 ▸), *SHELXS97* (Sheldrick, 2008 ▸), *SHELXL2014* (Sheldrick, 2015 ▸) and *ORTEP-3 for Windows* (Farrugia, 2012 ▸).

## supplementary crystallographic information

### Crystal data

**Table:**

[Ru(C_10_H_14_)(C_12_H_9_N_3_)(C_2_H_3_N)][SbF_6_]_2_	*D*_x_ = 2.078 Mg m^−^^3^
*M**_r_* = 943.06	Mo *K*α radiation, λ = 0.71073 Å
Orthorhombic, *P**b**c**a*	Cell parameters from 4920 reflections
*a* = 16.6046 (8) Å	θ = 1.8–25.2°
*b* = 15.5955 (7) Å	µ = 2.37 mm^−^^1^
*c* = 23.2786 (12) Å	*T* = 100 K
*V* = 6028.2 (5) Å^3^	Plate, green
*Z* = 8	0.18 × 0.17 × 0.08 mm
*F*(000) = 3616	

### Data collection

**Table:**

BrukerAPEXII with CCD diffractometer	6643 independent reflections
Radiation source: fine-focus sealed tube	4920 reflections with *I* > 2σ(*I*)
Graphite monochromator	*R*_int_ = 0.054
ω scans	θ_max_ = 27.2°, θ_min_ = 1.8°
Absorption correction: multi-scan (*SADABS*; Sheldrick, 1996)	*h* = −21→20
*T*_min_ = 0.578, *T*_max_ = 0.746	*k* = −19→19
27645 measured reflections	*l* = −29→27

### Refinement

**Table:**

Refinement on *F*^2^	0 restraints
Least-squares matrix: full	Hydrogen site location: inferred from neighbouring sites
*R*[*F*^2^ > 2σ(*F*^2^)] = 0.033	H-atom parameters constrained
*wR*(*F*^2^) = 0.074	*w* = 1/[σ^2^(*F*_o_^2^) + (0.0294*P*)^2^ + 2.8958*P*] where *P* = (*F*_o_^2^ + 2*F*_c_^2^)/3
*S* = 1.01	(Δ/σ)_max_ = 0.002
6643 reflections	Δρ_max_ = 0.89 e Å^−^^3^
392 parameters	Δρ_min_ = −1.00 e Å^−^^3^

### Special details

**Table:**

Experimental. X-ray crystallographic data for I were collected from a single-crystal sample, which was mounted on a loop fiber. Data were collected using a Bruker smart diffractometer equipped with an *APEX* II CCD Detector, a graphite monochromator. The crystal-to-detector distance was 5.0 cm, and the data collection was carried out in 512 *x* 512 pixel mode.
Geometry. All e.s.d.'s (except the e.s.d. in the dihedral angle between two l.s. planes) are estimated using the full covariance matrix. The cell e.s.d.'s are taken into account individually in the estimation of e.s.d.'s in distances, angles and torsion angles; correlations between e.s.d.'s in cell parameters are only used when they are defined by crystal symmetry. An approximate (isotropic) treatment of cell e.s.d.'s is used for estimating e.s.d.'s involving l.s. planes.
Refinement. Refinement of *F*^2^ against ALL reflections. The weighted *R*-factor *wR* and goodness of fit *S* are based on *F*^2^, conventional *R*-factors *R* are based on *F*, with *F* set to zero for negative *F*^2^. The threshold expression of *F*^2^ > σ(*F*^2^) is used only for calculating *R*-factors(gt) *etc*. and is not relevant to the choice of reflections for refinement. *R*-factors based on *F*^2^ are statistically about twice as large as those based on *F*, and *R*- factors based on ALL data will be even larger.

### Fractional atomic coordinates and isotropic or equivalent isotropic displacement parameters (Å^2^)

**Table:**

	*x*	*y*	*z*	*U*_iso_*/*U*_eq_	
Ru1	0.93384 (2)	0.22549 (2)	0.87058 (2)	0.01738 (8)	
Sb1	0.85442 (2)	−0.13417 (2)	0.94657 (2)	0.02309 (8)	
Sb2	0.78888 (2)	0.49530 (2)	0.71378 (2)	0.02768 (8)	
F1	0.89213 (19)	−0.09995 (16)	1.01877 (12)	0.0461 (8)	
F2	0.93350 (14)	−0.06576 (14)	0.91106 (11)	0.0282 (6)	
F3	0.77479 (15)	−0.20299 (16)	0.98028 (12)	0.0372 (7)	
F4	0.92598 (15)	−0.22620 (14)	0.94954 (12)	0.0359 (6)	
F5	0.82004 (18)	−0.1684 (2)	0.87420 (12)	0.0517 (8)	
F6	0.78500 (18)	−0.04062 (18)	0.94558 (15)	0.0605 (10)	
F7	0.7883 (2)	0.37969 (16)	0.69278 (13)	0.0558 (9)	
F8	0.7572 (2)	0.46434 (18)	0.78733 (12)	0.0538 (9)	
F9	0.79200 (19)	0.61114 (16)	0.73516 (12)	0.0464 (8)	
F10	0.6831 (2)	0.5081 (2)	0.69167 (18)	0.0795 (12)	
F11	0.89525 (19)	0.48324 (19)	0.73806 (18)	0.0732 (11)	
F12	0.8239 (3)	0.5243 (2)	0.64075 (14)	0.0872 (14)	
N1	0.90603 (19)	0.3372 (2)	0.96626 (14)	0.0178 (7)	
N2	0.8897 (2)	0.2522 (2)	1.04905 (14)	0.0214 (8)	
N3	0.91640 (18)	0.1923 (2)	0.95632 (14)	0.0176 (7)	
N5	1.0551 (2)	0.2291 (2)	0.88533 (14)	0.0210 (8)	
C2	0.9204 (2)	0.3380 (3)	0.90940 (18)	0.0206 (9)	
C3	0.9232 (3)	0.4290 (2)	0.89077 (17)	0.0223 (9)	
H3A	0.8806	0.4410	0.8621	0.027*	
H3B	0.9762	0.4431	0.8738	0.027*	
C4	0.9091 (2)	0.4796 (2)	0.94537 (18)	0.0214 (9)	
C5	0.9052 (3)	0.5662 (3)	0.95639 (19)	0.0263 (10)	
H5	0.9146	0.6068	0.9267	0.032*	
C6	0.8872 (3)	0.5931 (3)	1.0116 (2)	0.0281 (10)	
H6	0.8847	0.6528	1.0196	0.034*	
C7	0.8729 (3)	0.5347 (3)	1.05571 (19)	0.0267 (10)	
H7	0.8592	0.5549	1.0930	0.032*	
C8	0.8786 (2)	0.4467 (3)	1.04542 (18)	0.0223 (9)	
H8	0.8703	0.4058	1.0751	0.027*	
C9	0.8969 (2)	0.4222 (2)	0.99016 (18)	0.0191 (9)	
C10	0.9032 (2)	0.2573 (2)	0.99352 (18)	0.0190 (9)	
C11	0.8882 (2)	0.1724 (3)	1.07022 (18)	0.0230 (9)	
H11	0.8788	0.1650	1.1102	0.028*	
C12	0.8997 (2)	0.1006 (3)	1.03687 (17)	0.0229 (9)	
H12	0.8967	0.0446	1.0527	0.028*	
C13	0.9157 (2)	0.1136 (2)	0.97918 (18)	0.0219 (9)	
H13	0.9264	0.0655	0.9553	0.026*	
C27	1.1233 (3)	0.2267 (2)	0.88601 (17)	0.0209 (9)	
C28	1.2103 (3)	0.2249 (3)	0.8860 (2)	0.0326 (11)	
H28A	1.2303	0.2392	0.8476	0.049*	
H28B	1.2289	0.1674	0.8967	0.049*	
H28C	1.2307	0.2668	0.9138	0.049*	
C50	0.9606 (3)	0.2124 (3)	0.77680 (18)	0.0247 (10)	
C51	0.9519 (3)	0.1264 (3)	0.79863 (17)	0.0241 (10)	
H51	0.9932	0.0856	0.7916	0.029*	
C52	0.8850 (3)	0.1023 (3)	0.82936 (17)	0.0227 (9)	
H52	0.8808	0.0452	0.8433	0.027*	
C53	0.8216 (2)	0.1625 (3)	0.84050 (18)	0.0207 (9)	
C54	0.8274 (2)	0.2446 (2)	0.81594 (16)	0.0196 (9)	
H54	0.7842	0.2840	0.8205	0.024*	
C55	0.8962 (2)	0.2697 (3)	0.78465 (17)	0.0213 (9)	
H55	0.8990	0.3257	0.7687	0.026*	
C56	1.0360 (3)	0.2354 (3)	0.74384 (19)	0.0305 (11)	
H56	1.0823	0.2063	0.7631	0.037*	
C57	1.0281 (3)	0.1975 (3)	0.6834 (2)	0.0448 (14)	
H57A	1.0189	0.1355	0.6861	0.067*	
H57B	1.0777	0.2083	0.6617	0.067*	
H57C	0.9825	0.2244	0.6635	0.067*	
C58	1.0541 (3)	0.3316 (3)	0.7425 (2)	0.0445 (13)	
H58A	1.1071	0.3411	0.7252	0.067*	
H58C	1.0539	0.3542	0.7818	0.067*	
H58B	1.0129	0.3611	0.7198	0.067*	
C59	0.7499 (3)	0.1355 (3)	0.87511 (19)	0.0283 (10)	
H59C	0.7273	0.1855	0.8949	0.042*	
H59B	0.7664	0.0926	0.9035	0.042*	
H59A	0.7091	0.1108	0.8496	0.042*	

### Atomic displacement parameters (Å^2^)

**Table:**

	*U*^11^	*U*^22^	*U*^33^	*U*^12^	*U*^13^	*U*^23^
Ru1	0.01528 (16)	0.01805 (16)	0.01879 (17)	0.00165 (13)	0.00117 (14)	−0.00097 (14)
Sb1	0.01982 (15)	0.02391 (15)	0.02554 (16)	0.00207 (12)	0.00044 (13)	0.00291 (13)
Sb2	0.03160 (17)	0.02741 (16)	0.02404 (16)	0.00270 (13)	0.00236 (13)	−0.00066 (13)
F1	0.073 (2)	0.0358 (15)	0.0295 (16)	−0.0094 (15)	0.0018 (15)	−0.0053 (13)
F2	0.0254 (13)	0.0237 (12)	0.0354 (15)	−0.0027 (11)	−0.0018 (12)	0.0042 (11)
F3	0.0258 (15)	0.0391 (15)	0.0466 (17)	−0.0006 (12)	0.0117 (13)	0.0062 (13)
F4	0.0288 (15)	0.0234 (13)	0.0557 (18)	0.0042 (11)	0.0071 (13)	0.0046 (13)
F5	0.0488 (19)	0.075 (2)	0.0315 (17)	−0.0302 (17)	−0.0106 (14)	0.0072 (15)
F6	0.0386 (18)	0.0464 (17)	0.096 (3)	0.0217 (14)	0.0235 (18)	0.0285 (18)
F7	0.089 (3)	0.0309 (16)	0.0481 (19)	0.0035 (15)	0.0041 (18)	−0.0131 (14)
F8	0.082 (2)	0.0438 (17)	0.0351 (17)	−0.0126 (17)	0.0162 (16)	0.0004 (14)
F9	0.068 (2)	0.0297 (15)	0.0410 (17)	−0.0018 (14)	0.0026 (16)	0.0015 (13)
F10	0.055 (2)	0.085 (3)	0.098 (3)	0.011 (2)	−0.041 (2)	−0.017 (2)
F11	0.0317 (18)	0.0531 (19)	0.135 (4)	0.0016 (15)	−0.007 (2)	0.019 (2)
F12	0.164 (4)	0.055 (2)	0.042 (2)	0.028 (2)	0.048 (2)	0.0118 (17)
N1	0.0178 (17)	0.0198 (17)	0.0158 (18)	0.0006 (14)	0.0008 (14)	−0.0036 (14)
N2	0.0171 (18)	0.0243 (19)	0.023 (2)	0.0003 (15)	−0.0010 (15)	−0.0002 (16)
N3	0.0131 (17)	0.0188 (17)	0.0209 (19)	0.0016 (13)	0.0025 (14)	−0.0020 (15)
N5	0.023 (2)	0.0222 (18)	0.0182 (19)	0.0017 (15)	0.0006 (15)	−0.0003 (15)
C2	0.010 (2)	0.027 (2)	0.024 (2)	0.0011 (16)	−0.0018 (17)	−0.0022 (18)
C3	0.025 (2)	0.025 (2)	0.017 (2)	−0.0011 (18)	0.0005 (18)	0.0039 (18)
C4	0.014 (2)	0.023 (2)	0.027 (2)	0.0000 (16)	−0.0017 (18)	−0.0017 (19)
C5	0.022 (2)	0.027 (2)	0.030 (3)	0.0025 (18)	−0.0022 (19)	0.003 (2)
C6	0.024 (2)	0.025 (2)	0.035 (3)	0.0022 (19)	−0.002 (2)	−0.009 (2)
C7	0.025 (2)	0.028 (2)	0.027 (2)	−0.0029 (19)	0.002 (2)	−0.008 (2)
C8	0.020 (2)	0.022 (2)	0.024 (2)	−0.0023 (17)	0.0005 (18)	−0.0019 (19)
C9	0.013 (2)	0.019 (2)	0.025 (2)	−0.0008 (16)	−0.0015 (17)	−0.0016 (18)
C10	0.0097 (19)	0.025 (2)	0.022 (2)	−0.0012 (16)	−0.0028 (17)	−0.0017 (18)
C11	0.020 (2)	0.030 (2)	0.020 (2)	0.0007 (18)	−0.0028 (18)	0.0031 (19)
C12	0.022 (2)	0.027 (2)	0.020 (2)	−0.0031 (18)	−0.0039 (18)	0.0056 (19)
C13	0.020 (2)	0.020 (2)	0.026 (2)	0.0012 (16)	−0.0060 (19)	−0.0006 (18)
C27	0.026 (2)	0.020 (2)	0.017 (2)	−0.0017 (18)	0.0019 (18)	−0.0029 (17)
C28	0.020 (2)	0.043 (3)	0.035 (3)	−0.005 (2)	0.002 (2)	−0.007 (2)
C50	0.024 (2)	0.032 (2)	0.018 (2)	0.0057 (19)	−0.0021 (18)	−0.0047 (19)
C51	0.026 (2)	0.027 (2)	0.019 (2)	0.0100 (18)	−0.0035 (18)	−0.0101 (19)
C52	0.027 (2)	0.022 (2)	0.020 (2)	−0.0010 (18)	−0.0037 (19)	−0.0070 (18)
C53	0.015 (2)	0.025 (2)	0.022 (2)	0.0001 (17)	−0.0028 (18)	−0.0043 (18)
C54	0.016 (2)	0.025 (2)	0.017 (2)	0.0041 (17)	−0.0047 (17)	−0.0043 (18)
C55	0.024 (2)	0.024 (2)	0.016 (2)	0.0022 (18)	−0.0005 (18)	0.0024 (18)
C56	0.026 (2)	0.043 (3)	0.022 (2)	0.005 (2)	0.007 (2)	0.003 (2)
C57	0.045 (3)	0.066 (4)	0.023 (3)	0.003 (3)	0.014 (2)	0.001 (3)
C58	0.036 (3)	0.053 (3)	0.045 (3)	−0.004 (3)	0.013 (3)	0.001 (3)
C59	0.026 (2)	0.028 (2)	0.031 (3)	−0.006 (2)	−0.001 (2)	−0.001 (2)

### Geometric parameters (Å, º)

**Table:**

Ru1—C2	1.986 (4)	C56—C57	1.533 (6)
Ru1—N3	2.082 (3)	C56—H56	1.0000
Ru1—N5	2.044 (3)	C57—H57A	0.9800
Ru1—C50	2.237 (4)	C57—H57B	0.9800
Ru1—C51	2.298 (4)	C57—H57C	0.9800
Ru1—C52	2.296 (4)	C58—H58A	0.9800
Ru1—C53	2.220 (4)	C58—H58C	0.9800
Ru1—C54	2.197 (4)	C58—H58B	0.9800
Ru1—C55	2.206 (4)	C2—N1	1.345 (5)
Sb1—F1	1.871 (3)	C2—C3	1.485 (5)
Sb1—F2	1.883 (2)	C3—C4	1.514 (5)
Sb1—F3	1.875 (2)	C3—H3A	0.9900
Sb1—F4	1.865 (2)	C3—H3B	0.9900
Sb1—F5	1.857 (3)	C4—C5	1.377 (6)
Sb1—F6	1.860 (3)	C4—C9	1.388 (5)
Sb2—F7	1.868 (3)	C9—C8	1.376 (5)
Sb2—F8	1.855 (3)	C9—N1	1.447 (5)
Sb2—F9	1.875 (3)	C8—C7	1.396 (6)
Sb2—F10	1.841 (3)	C8—H8	0.9500
Sb2—F11	1.864 (3)	C7—C6	1.393 (6)
Sb2—F12	1.853 (3)	C7—H7	0.9500
N5—C27	1.133 (5)	C6—C5	1.384 (6)
C50—C55	1.406 (6)	C6—H6	0.9500
C50—C51	1.442 (6)	C5—H5	0.9500
C50—C56	1.510 (6)	N1—C10	1.399 (5)
C51—C52	1.373 (6)	C10—N2	1.314 (5)
C51—H51	0.9500	C10—N3	1.351 (5)
C52—C53	1.436 (5)	N2—C11	1.339 (5)
C52—H52	0.9500	C11—C12	1.375 (6)
C53—C54	1.406 (5)	C11—H11	0.9500
C53—C59	1.497 (6)	C12—C13	1.384 (6)
C54—C55	1.409 (6)	C12—H12	0.9500
C54—H54	0.9500	C13—N3	1.338 (5)
C55—H55	0.9500	C13—H13	0.9500
C59—H59C	0.9800	C27—C28	1.444 (6)
C59—H59B	0.9800	C28—H28A	0.9800
C59—H59A	0.9800	C28—H28B	0.9800
C56—C58	1.531 (6)	C28—H28C	0.9800
			
C2—Ru1—N5	90.54 (14)	C59—C53—Ru1	128.4 (3)
C2—Ru1—N3	76.59 (15)	C53—C54—C55	121.3 (4)
N5—Ru1—N3	89.02 (13)	C53—C54—Ru1	72.3 (2)
C2—Ru1—C54	93.05 (15)	C55—C54—Ru1	71.7 (2)
N5—Ru1—C54	152.34 (14)	C53—C54—H54	119.4
N3—Ru1—C54	118.48 (14)	C55—C54—H54	119.4
C2—Ru1—C55	96.00 (16)	Ru1—C54—H54	129.0
N5—Ru1—C55	115.03 (14)	C50—C55—C54	120.5 (4)
N3—Ru1—C55	155.13 (14)	C50—C55—Ru1	72.7 (2)
C54—Ru1—C55	37.32 (15)	C54—C55—Ru1	71.0 (2)
C2—Ru1—C53	116.11 (15)	C50—C55—H55	119.8
N5—Ru1—C53	153.20 (14)	C54—C55—H55	119.8
N3—Ru1—C53	94.33 (14)	Ru1—C55—H55	128.8
C54—Ru1—C53	37.11 (14)	C53—C59—H59C	109.5
C55—Ru1—C53	67.31 (15)	C53—C59—H59B	109.5
C2—Ru1—C50	123.14 (16)	H59C—C59—H59B	109.5
N5—Ru1—C50	88.31 (14)	C53—C59—H59A	109.5
N3—Ru1—C50	160.11 (14)	H59C—C59—H59A	109.5
C54—Ru1—C50	66.88 (15)	H59B—C59—H59A	109.5
C55—Ru1—C50	36.89 (14)	C50—C56—C58	113.9 (4)
C53—Ru1—C50	79.57 (15)	C50—C56—C57	107.7 (4)
C2—Ru1—C52	152.89 (15)	C58—C56—C57	112.1 (4)
N5—Ru1—C52	116.18 (14)	C50—C56—H56	107.6
N3—Ru1—C52	98.24 (14)	C58—C56—H56	107.6
C54—Ru1—C52	65.67 (15)	C57—C56—H56	107.6
C55—Ru1—C52	77.44 (15)	C56—C57—H57A	109.5
C53—Ru1—C52	37.03 (14)	C56—C57—H57B	109.5
C50—Ru1—C52	65.55 (15)	H57A—C57—H57B	109.5
C2—Ru1—C51	160.05 (16)	C56—C57—H57C	109.5
N5—Ru1—C51	90.72 (14)	H57A—C57—H57C	109.5
N3—Ru1—C51	123.34 (14)	H57B—C57—H57C	109.5
C54—Ru1—C51	76.96 (15)	C56—C58—H58A	109.5
C55—Ru1—C51	65.56 (15)	C56—C58—H58C	109.5
C53—Ru1—C51	65.32 (15)	H58A—C58—H58C	109.5
C50—Ru1—C51	37.03 (15)	C56—C58—H58B	109.5
C52—Ru1—C51	34.79 (15)	H58A—C58—H58B	109.5
F5—Sb1—F6	91.35 (15)	H58C—C58—H58B	109.5
F5—Sb1—F4	90.48 (13)	N1—C2—C3	107.6 (3)
F6—Sb1—F4	178.08 (15)	N1—C2—Ru1	117.3 (3)
F5—Sb1—F1	178.35 (14)	C3—C2—Ru1	135.1 (3)
F6—Sb1—F1	89.71 (14)	C2—C3—C4	104.4 (3)
F4—Sb1—F1	88.45 (12)	C2—C3—H3A	110.9
F5—Sb1—F3	89.91 (12)	C4—C3—H3A	110.9
F6—Sb1—F3	90.98 (12)	C2—C3—H3B	110.9
F4—Sb1—F3	89.61 (11)	C4—C3—H3B	110.9
F1—Sb1—F3	91.34 (12)	H3A—C3—H3B	108.9
F5—Sb1—F2	88.79 (11)	C5—C4—C9	119.0 (4)
F6—Sb1—F2	88.99 (11)	C5—C4—C3	132.5 (4)
F4—Sb1—F2	90.46 (10)	C9—C4—C3	108.5 (3)
F1—Sb1—F2	89.96 (12)	C8—C9—C4	123.8 (4)
F3—Sb1—F2	178.70 (12)	C8—C9—N1	129.6 (4)
F10—Sb2—F12	91.0 (2)	C4—C9—N1	106.7 (3)
F10—Sb2—F8	90.89 (17)	C9—C8—C7	116.6 (4)
F12—Sb2—F8	178.02 (18)	C9—C8—H8	121.7
F10—Sb2—F11	178.52 (18)	C7—C8—H8	121.7
F12—Sb2—F11	90.30 (19)	C6—C7—C8	120.3 (4)
F8—Sb2—F11	87.86 (17)	C6—C7—H7	119.8
F10—Sb2—F7	91.56 (15)	C8—C7—H7	119.8
F12—Sb2—F7	89.85 (14)	C5—C6—C7	121.5 (4)
F8—Sb2—F7	89.37 (13)	C5—C6—H6	119.2
F11—Sb2—F7	89.24 (15)	C7—C6—H6	119.2
F10—Sb2—F9	89.76 (14)	C4—C5—C6	118.7 (4)
F12—Sb2—F9	89.97 (14)	C4—C5—H5	120.6
F8—Sb2—F9	90.77 (12)	C6—C5—H5	120.6
F11—Sb2—F9	89.45 (14)	C2—N1—C10	117.4 (3)
F7—Sb2—F9	178.67 (15)	C2—N1—C9	112.8 (3)
C27—N5—Ru1	170.4 (3)	C10—N1—C9	129.7 (3)
C55—C50—C51	117.9 (4)	N2—C10—N3	127.8 (4)
C55—C50—C56	123.1 (4)	N2—C10—N1	120.4 (4)
C51—C50—C56	118.9 (4)	N3—C10—N1	111.8 (3)
C55—C50—Ru1	70.4 (2)	C10—N2—C11	114.9 (4)
C51—C50—Ru1	73.8 (2)	N2—C11—C12	123.1 (4)
C56—C50—Ru1	129.7 (3)	N2—C11—H11	118.5
C52—C51—C50	121.4 (4)	C12—C11—H11	118.5
C52—C51—Ru1	72.5 (2)	C11—C12—C13	117.1 (4)
C50—C51—Ru1	69.2 (2)	C11—C12—H12	121.4
C52—C51—H51	119.3	C13—C12—H12	121.4
C50—C51—H51	119.3	N3—C13—C12	121.4 (4)
Ru1—C51—H51	132.0	N3—C13—H13	119.3
C51—C52—C53	120.5 (4)	C12—C13—H13	119.3
C51—C52—Ru1	72.7 (2)	C13—N3—C10	115.6 (3)
C53—C52—Ru1	68.6 (2)	C13—N3—Ru1	127.6 (3)
C51—C52—H52	119.7	C10—N3—Ru1	116.8 (3)
C53—C52—H52	119.7	N5—C27—C28	178.9 (5)
Ru1—C52—H52	131.8	C27—C28—H28A	109.5
C54—C53—C52	118.2 (4)	C27—C28—H28B	109.5
C54—C53—C59	122.0 (4)	H28A—C28—H28B	109.5
C52—C53—C59	119.8 (4)	C27—C28—H28C	109.5
C54—C53—Ru1	70.6 (2)	H28A—C28—H28C	109.5
C52—C53—Ru1	74.3 (2)	H28B—C28—H28C	109.5

### Hydrogen-bond geometry (Å, º)

**Table:**

*D*—H···*A*	*D*—H	H···*A*	*D*···*A*	*D*—H···*A*
C11—H11···F7^i^	0.95	2.54	3.398 (6)	151
C12—H12···F1	0.95	2.39	3.157 (5)	138
C13—H13···F2	0.95	2.30	3.229 (5)	167
C51—H51···F11^ii^	0.95	2.54	3.485 (6)	174
C52—H52···F2	0.95	2.50	3.337 (5)	147
C54—H54···F5^iii^	0.95	2.26	3.110 (5)	148
C59—H59*B*···F6	0.98	2.32	3.253 (6)	158
C59—H59*C*···F5^iii^	0.98	2.45	3.270 (6)	141

Symmetry codes: (i) *x*, −*y*+1/2, *z*+1/2; (ii) −*x*+2, *y*−1/2, −*z*+3/2; (iii) −*x*+3/2, *y*+1/2, *z*.

## References

[bb1] Ackermann, L. & Lygin, A. V. (2011). *Org. Lett.* **13**, 3332–3335. 10.1021/ol2010648 21644545

[bb2] Arockiam, P. B., Bruneau, C. & Dixneuf, P. H. (2012). *Chem. Rev.* **112**, 5879–5918. 10.1021/cr300153j 22934619

[bb3] Bruker (2012). *APEX2* and *SAINT*. Bruker AXS Inc. Madison, Wisconsin, USA.

[bb4] Buil, M. L., Esteruelas, M. A., López, A. M. & Oñate, E. (2003). *Organometallics*, **22**, 5274–5284.

[bb5] Chiang, P.-Y., Lin, Y.-C., Wang, Y. & Liu, Y.-H. (2010). *Organomet­allics*, **29**, 5776–5782.

[bb6] Farrugia, L. J. (2012). *J. Appl. Cryst.* **45**, 849–854.

[bb7] Ferrer Flegeau, E., Bruneau, C., Dixneuf, P. H. & Jutand, A. (2011). *J. Am. Chem. Soc.* **133**, 10161–10170. 10.1021/ja201462n 21604765

[bb8] Li, B., Roisnel, T., Darcel, C. & Dixneuf, P. H. (2012). *Dalton Trans.* **41**, 10934–10937. 10.1039/c2dt31401k 22890507

[bb9] Sheldrick, G. M. (1996). *SADABS*. University of Göttingen, Germany.

[bb10] Sheldrick, G. M. (2008). *Acta Cryst.* A**64**, 112–122. 10.1107/S0108767307043930 18156677

[bb11] Sheldrick, G. M. (2015). *Acta Cryst.* C**71**, 3–8.

[bb12] Sollert, C., Devaraj, K., Orthaber, A., Gates, P. J. & Pilarski, L. T. (2015). *Chem. Eur. J.* **21**, 5380–5386. 10.1002/chem.201405931 PMC4600241 25689052

[bb13] Wang, L., Yang, D., Han, F., Li, D., Zhao, D. & Wang, R. (2015). *Org. Lett.* **17**, 176–179. 10.1021/ol503455r 25551662

